# Traditional medicine practices among community members with chronic kidney disease in northern Tanzania: an ethnomedical survey

**DOI:** 10.1186/s12882-015-0161-y

**Published:** 2015-10-23

**Authors:** John W. Stanifer, Joseph Lunyera, David Boyd, Francis Karia, Venance Maro, Justin Omolo, Uptal D. Patel

**Affiliations:** Department of Medicine, Duke University, Durham, NC USA; Duke Global Health Institute, Duke University, Durham, NC USA; Kilimanjaro Christian Medical College, Moshi, Tanzania Africa; National Institute for Medical Research, Dar es Salaam, Tanzania Africa; Duke Clinical Research Institute, Duke University, Durham, NC USA

**Keywords:** Epidemiology, Ethnopharmacology, Herbal medicine, Non-communicable diseases, Sub-Saharan Africa

## Abstract

**Background:**

In sub-Saharan Africa, chronic kidney disease (CKD) is being recognized as a non-communicable disease (NCD) with high morbidity and mortality. In countries like Tanzania, people access many sources, including traditional medicines, to meet their healthcare needs for NCDs, but little is known about traditional medicine practices among people with CKD. Therefore, we sought to characterize these practices among community members with CKD in northern Tanzania.

**Methods:**

Between December 2013 and June 2014, we administered a previously-developed survey to a random sample of adult community-members from the Kilimanjaro Region; the survey was designed to measure traditional medicine practices such as types, frequencies, reasons, and modes. Participants were also tested for CKD, diabetes, hypertension, and HIV as part of the CKD-AFRiKA study. To identify traditional medicines used in the local treatment of kidney disease, we reviewed the qualitative sessions which had previously been conducted with key informants.

**Results:**

We enrolled 481 adults of whom 57 (11.9 %) had CKD. The prevalence of traditional medicine use among adults with CKD was 70.3 % (95 % CI 50.0–84.9 %), and among those at risk for CKD (*n* = 147; 30.6 %), it was 49.0 % (95 % CI 33.1–65.0 %). Among adults with CKD, the prevalence of concurrent use of traditional medicine and biomedicine was 33.2 % (11.4–65.6 %). Symptomatic ailments (66.7 %; 95 % CI 17.3–54.3), malaria/febrile illnesses (64.0 %; 95 % CI 44.1–79.9), and chronic diseases (49.6 %; 95 % CI 28.6–70.6) were the most prevalent uses for traditional medicines. We identified five plant–based traditional medicines used for the treatment of kidney disease: *Aloe vera*, *Commifora africana*, *Cymbopogon citrullus*, *Persea americana*, and *Zanthoxylum chalybeum*.

**Conclusions:**

The prevalence of traditional medicine use is high among adults with and at risk for CKD in northern Tanzania where they use them for a variety of conditions including other NCDs. Additionally, many of these same people access biomedicine and traditional medicines concurrently. The traditional medicines used for the local treatment of kidney disease have a variety of activities, and people with CKD may be particularly vulnerable to adverse effects. Recognizing these traditional medicine practices will be important in shaping CKD treatment programs and public health policies aimed at addressing CKD.

**Electronic supplementary material:**

The online version of this article (doi:10.1186/s12882-015-0161-y) contains supplementary material, which is available to authorized users.

## Background

Non-communicable diseases (NCDs) are a growing burden in sub-Saharan Africa with significant and disproportionate morbidity and mortality [1,2]. Among the NCDs in sub-Saharan Africa, chronic kidney disease (CKD) is being recognized as a disease with a high prevalence and high morbidity and mortality [3]. In Tanzania, the prevalence is estimated to be 7 % of the population with as many as 15 % of adults in urban settings living with CKD, and despite this high prevalence, awareness is low [4].

In Tanzania, people access a variety of resources to meet their healthcare needs, and at least 60 % of the population is estimated to use traditional medicines (TMs) [5]. TMs are used for numerous conditions and ailments including NCDs; however, their use among community members with CKD is not well-known. In similar sub-Saharan African settings, TMs have been associated with acute and chronic kidney injury, and their use may positively or negatively impact the effectiveness of interventions geared toward CKD [5–7]. As such, characterization of TM use and practices is an important step in formulating disease management programs as well as informing optimal public health efforts aimed at addressing the significant regional CKD burden.

The Comprehensive Kidney Disease Assessment for Risk Factors, epidemiology, Knowledge, and Attitudes (CKD-AFRiKA) study is an ongoing project in northern Tanzania with the goal of understanding the epidemiology, etiology, knowledge, attitudes and practices associated with CKD as well as other related NCDs. As part of the study, we conducted assessments that included focus group discussions (FGDs), in-depth interviews, and the administration of a structured survey to community-based adults. Our overall objective was to characterize TM use among a community-based population so that we may better inform local biomedical healthcare practices and help shape public health efforts that are sensitive to TM practices. Our specific aims were to explore the practices, including types, frequencies, reasons, and modes of TMs used, among community-members with and at risk for CKD.

## Methods

### Ethics, consent, and permissions

The study protocol was approved by Duke University Institutional Review Board (#Pro00040784), the Kilimanjaro Christian Medical College (KCMC) Ethics Committee (EC#502), and the National Institute for Medical Research in Tanzania. The consent forms were administered verbally to all participants, and written informed consent (by signature or thumbprint) was obtained from all participants.

### Study setting

The CKD AFRIKA Study was conducted between December 2013 and June 2014 in the Kilimanjaro Region of Tanzania. The region has an adult population of 900,000 many of whom (35 %) live in an urban setting. The HIV prevalence is 3–5 %. The unemployment rate is 19 %, and the majority of adults have a primary education or less (77 %). The largest ethnic group is the Chagga tribe followed by the Pare, Sambaa, and Maasai tribes [8]. Swahili is the major language, and all participants in our study spoke it as their first language.

### Quantitative data collection

We developed a structured survey instrument designed to test different factors related to TM practices among community members (Additional file 1: Appendix 1). The development of this survey has been described elsewhere, but in brief, the instrument was drafted by local and non-local experts from multiple disciplines including medicine, epidemiology, sociology, anthropology, and public health. It was independently translated into Swahili by two native speakers, and we conducted joint reviews of each version with a focus on the codability of words and concepts with difficult translations. To ensure the content validity of the survey instrument, we piloted it through multiple qualitative sessions. This was an iterative process that involved several adjustments to the instrument as new themes and ideas emerged throughout the sessions. Many of the survey items and response categories were added directly based on the results of these qualitative piloting sessions [5]. In its final form, the survey instrument included nine items. It comprised open-ended questions related to types of TMs used by community members as well as close-ended questions related to frequency of use, reasons for use, modes of use, modes of access, and conditions treated by TMs.

Using two local surveyors, we verbally administered the survey to adult community members stratified by urban and rural status. Using a random-number generator, we selected thirty-seven sampling areas from twenty-nine neighborhoods within the Moshi Urban and Moshi Rural districts. We based the random neighborhood selection on probability proportional to size using the 2012 Tanzanian National Census [8]. Within each neighborhood the sampling area was determined using geographic points randomly generated using Arc Global Information Systems (ArcGIS), v10.2.2 (Environmental Systems Research Institute, Redlands, CA). Households were then randomly chosen based on coin-flip and die-rolling techniques according to a pre-established protocol [4]. We targeted an enrollment between 15 and 25 participants per sampling area, but the overall sample size was based on the requirements of the CKD AFRIKA study which was designed to estimate the community prevalence of CKD with a precision of 5 % when accounting for the cluster-design effect.

All adults living in the selected households were recruited, and all Tanzanian citizens over the age of 18 were eligible for inclusion. To reduce non-response rates, we attempted a minimum of two additional visits on subsequent days and weekends, and using mobile phone numbers, we located eligible participants through multiple phone calls. Additionally, when available, we collected demographic data including gender, age, and occupation for the non-responders.

### Qualitative data collection

As part of the CKD AFRIKA project, FGDs and in-depth interviews were conducted in a central, easily accessible area to the participants. The methodological details of these sessions have been described elsewhere, but in brief, we conducted five FGDs and 27 in-depth interviews both of which included key informants from the community including well-adults from the general population, chronically-ill adults receiving care at the hospital medicine clinics, adults receiving care from traditional healers, adults purchasing TMs from herbal vendors, traditional healers, herbal vendors, and medical doctors [5].

### Disease definitions

Participants who completed a structured survey were also tested for CKD, diabetes, hypertension, and human immunodeficiency virus (HIV) as part of the CKD AFRIKA Study [4]. CKD was defined as the presence of albuminuria (≥30 mg/dL; confirmed by repeat assessment) and/or a reduction in the estimated glomerular filtration rate (eGFR) ≤60 ml/min/1.73 m^2^ according to the Modification of Diet in Renal Disease equation without the race factor [9]. Hypertension was defined as a single blood pressure measurement of greater than 160/100 mmHg, a two-measurement average greater than 140/90 mmHg, or the ongoing use of anti-hypertensive medications. Diabetes was defined as a hemoglobin A1c (HbA1c) level ≥ 7.0 % or the ongoing use of anti-hyperglycemic medications, and HIV was defined as a positive Alere Determine HIV −1/2 assay (Alere Medical Co. Ltd; Waltham, MA) confirmed by a Uni-Gold HIV assay (Trinity Biotech Manufacturing Ltd; Wicklow, Ireland), a self-reported history, or the ongoing use of highly active anti-retroviral therapy.

We considered participants to be at risk for CKD if they had poorly controlled diabetes, hypertension, or HIV. We considered diabetes to be poorly controlled if the HbA1c level was ≥7.0 % with or without biomedical therapy; we considered hypertension to be poorly controlled if the blood pressure was ≥140/90 mmHg on two-time average or ≥160/100 mmHg on one-time measurement. We defined poorly controlled HIV as being positive for HIV but not currently receiving biomedical care.

### Data analysis and management

Quantitative data were analyzed using STATAv.13 (STATA Corp., College Station, TX). The median and inter-quartile ranges (IQR) were reported for continuous variables. All p-values are two-sided at a 0.05 significance level. To compare differences between groups, we used a Chi squared test, Fisher’s Exact test, or the Wilcoxon-Mann–Whitney rank sum test. Prevalence estimates were sample-balanced using age- and gender-weights based on the 2012 urban and rural district-level census data [8]. Prevalence ratios (PR) were estimated using generalized linear models assuming a log link, and separate univariable models were fitted to the outcome for each variable including gender, age, ethnicity, education, setting (urban or rural), occupation, and self-reported medical histories of HIV or an NCD including diabetes, hypertension, stroke, heart disease, or chronic obstructive lung disease/asthma. We used Taylor Series linearization to account for the design effect due to cluster sampling. All data were collected on paper and then electronically entered into a purpose-built Research Electronic Data Capture (REDCap) database. All data were verified after electronic data entry by an independent reviewer to ensure accuracy.

To identify traditional medicines used for the treatment of kidney disease, two authors (JWS and JL) independently reviewed the transcripts of the FGDs and in-depth interviews conducted as part of the CKD AFRIKA Study. All traditional medicines referenced by participants were recorded in the coding index, and analytic memos were created for traditional medicines referenced as part of treatment for kidney diseases. A third author (JO) familiar with local languages, dialects, and customs, then cross-referenced the local vernacular with known botanical research catalogues in the region and country. Any discrepancies were resolved by joint consensus. All of the qualitative data, including the traditional medicine coding index, were recorded and managed using NViVOv.10.0 (QRS International Pty Ltd, Melbourne, Australia).

## Results

### Demographics

We enrolled 481 adults in the CKD AFRIKA Study of whom 57 (11.9 %) had CKD. The household non-response rate was 15.0 % and the individual non-response rate was 21 %. Inability to locate or contact individuals was the most common reason for non-response. Men (*p* < 0.001) and adults 18–39 years old (*p* = 0.001) were more likely to be non-responders, and the proportion of participants with a secondary or post-secondary education (22 %) was slightly higher than the expected regional average (15 %) (*p* = 0.02).

Most of the participants with CKD were urban residents (*n* = 54; 95 %), female (*n* = 40; 70 %), ethnically Chagga (n = 37; 65 %), had a primary school education (*n* = 40; 70 %) and worked in a self-employed small business/vendor (*n* = 21; 37 %) (Table 1). The median age of CKD participants was 45 years (IQR: 35–59), and many reported a history of hypertension (*n* = 25; 44 %), diabetes (*n* = 17; 30 %), heart disease (*n* = 6; 11 %) and HIV (n = 6; 11 %). Few participants with CKD were aware of their condition (*n* = 6; 11 %). Most adults with CKD had a history of alcohol intake (*n* = 41; 72 %), and a few reported a history of smoking (*n* = 17; 30 %). We also identified 147 (31 %) participants who did not have CKD but were considered to be at increased risk for it. Among these participants, 123 (84 %) had poorly controlled hypertension, 28 (19 %) had poorly controlled diabetes, and 8 (5.4 %) had poorly controlled HIV. Similar to those participants with CKD (all p values > 0.05), these high-risk participants were also mostly female (*n* = 102; 69 %), ethnically Chagga (*n* = 99; 67 %), and had a primary school level of education (*n* = 99; 67 %); however, they were more likely to be rural (*n* = 38; 26 %) compared to those with CKD (*p* < 0.01), and they were occupied most frequently as farmers (*n* = 66; 45 %) (*p* < 0.01).

**Table 1:** Participant characteristics by CKD and CKD Risk Status; CKD-AFRIKA study population, 2014; *N* = 481

Variable	Participants				
	Overall (*n* = 481)	CKD Present (*n* = 57)	CKD Absent (*n* = 424)		*p*-value^a^
			Low risk (*n* = 277)	Increased risk (*n* = 147)	
Gender					0.43
Male	123 (26 %)	17 (30 %)	61 (22 %)	45 (31 %)	
Female	358 (74 %)	40 (70 %)	216 (78 %)	102 (69 %)	
Age					0.39
18-39 years old	172 (36 %)	16 (28 %)	128 (46 %)	28 (19 %)	
40–59 years old	191 (40 %)	24 (42 %)	112 (40 %)	55 (37 %)	
60+ years old	118 (24 %)	17 (30 %)	37 (14 %)	64 (44 %)	
Setting					<0.01
Rural	111 (23 %)	3 (5 %)	70 (25 %)	38 (26 %)	
Urban	370 (77 %)	54 (95 %)	207 (75 %)	109 (74 %)	
Ethnicity					0.81
Chagga	288 (60 %)	37 (65 %)	152 (55 %)	99 (67 %)	
Pare	66 (14 %)	7 (12 %)	47 (17 %)	12 (8 %)	
Sambaa	27 (6 %)	4 (7 %)	19 (7 %)	4 (3 %)	
Other^b^	100 (20 %)	9 (16 %)	59 (21 %)	32 (22 %)	
Education					0.35
None	31 (6 %)	4 (7 %)	7 (3 %)	20 (14 %)	
Primary	349 (73 %)	40 (70 %)	210 (76 %)	99 (67 %)	
Secondary	74 (15 %)	7 (12 %)	47 (17 %)	20 (14 %)	
Post–Secondary	27 (6 %)	6 (11 %)	13 (5 %)	8 (5 %)	
Occupation					<0.01
Unemployed	74 (15 %)	10 (17 %)	47 (17 %)	17 (12 %)	
Farmer/Wage Earner	199 (41 %)	14 (25 %)	119 (43 %)	66 (45 %)	
Small Business/Vendors	158 (33 %)	21 (37 %)	100 (36 %)	37 (25 %)	
Professional^c^	50 (10 %)	12 (21 %)	11 (4 %)	27 (18 %)	
History of Smoking	117 (24 %)	16 (28 %)	61 (22 %)	40 (27 %)	0.44
History of alcohol intake	318 (66 %)	40 (70 %)	166 (60 %)	112 (76 %)	0.75
Self-Reported Medical History					
Hypertension	134 (28 %)	25 (44 %)	51 (19 %)	58 (39 %)	<0.01
Diabetes	61 (13 %)	17 (30 %)	15 (5 %)	29 (20 %)	<0.01
Heart Disease^d^	18 (4 %)	6 (11 %)	5 (2 %)	7 (5 %)	<0.01
HIV	21 (4 %)	6 (11 %)	5 (2 %)	10 (7 %)	0.02
Stroke	8 (2 %)	2 (4 %)	2 (1 %)	4 (3 %)	0.25
COPD	25 (5 %)	0 (0 %)	4 (1 %)	4 (3 %)	0.30
Kidney Disease	14 (3 %)	6 (11 %)	6 (2 %)	2 (1 %)	<0.01

^a^P-value comparing differences by CKD status (present or absent)

^b^Other ethnicities includes Maasai, Luguru, Kilindi, Kurya, Mziguwa, Mnyisanzu, Rangi, Jita, Nyambo, and Kaguru

^c^Professional included any salaried position (e.g. nurse, teacher, government employee, etc.) and retired persons

^d^Heart disease included coronary disease, heart failure, or structural diseases

COPD = Chronic obstructive pulmonary disease

### Epidemiology of traditional medicine use

TM use was high among participants with CKD. The prevalence of TM use was 70.3 % (95 % confidence interval [CI] 50.0-84.9 %) with most adults using TMs 1–5 times (54.4 %; 95 % CI 33.4–73.9 %) or 6–10 times (11.7 %; 95 % CI 3.30–33.6 %) per year (Table 2). The incidence of TM use of more than ten times per year was 4.30 % (95 % CI 0.01–20.7 %). Among adults with CKD, the prevalence of concurrent TM and biomedicine use was 33.2 % (95 % CI 11.4–65.6 %). In univariable regression, there was no significant difference in TM use by age, gender, ethnicity, setting (urban/rural), self-reported history of an NCD or HIV, or the ongoing use of biomedicines. Having obtained only a primary level of education (PR 6.42; *p* = 0.07) or working in a professional occupation (PR 3.95; *p* = 0.07) had the strongest associations with TM use among adults with CKD. When compared to biomedicines, the prevalence of community members with CKD reporting TMs as more effective was 85.4 % (95 % CI 65.3–94.8), as having a lower cost was 65.7 % (95 % CI 45.2–81.7), of being easier to access was 58.7 % (95 % CI 39.3–75.6), as being safer was 54.9 % (95 % CI 34.3–74.0), and as being more traditional or religious was 37.9 % (95 % CI 21.1–58.0).

**Table 2:** Epidemiology and characteristics of traditional medicine (TM) use stratified by CKD and CKD risk status; CKD-AFRIKA (2014)

	With CKD; *n* = 57	CKD Absent; *n* = 424	
	(%, 95 % CI)	(%, 95 % CI)	
		Low risk (*n* = 277)	Increased risk (*n* = 147)
Prevalence			
of TM Use	70.3 % (50.0–84.9)	65.3 % (55.2–74.1)	49.0 % (33.1–65.0)
of concurrent TM and Biomedicine Use	33.2 % (11.4–65.6)	4.35 % (2.15–8.61)	10.4 % (4.70–21.6)
Incidence of TM Use (per year)			
1–5 times	54.4 % (33.4–73.9)	50.5 % (41.3–59.6)	31.0 % (20.8–43.3)
6–10 times	11.7 % (3.30–33.6)	7.69 % (4.86–12.0)	11.5 % (5.50–22.4)
>10 times	4.30 % (0.01–20.7)	7.07 % (3.78–12.8)	7.10 % (3.60–13.5)
Reasons for TM Use			
More Effective	85.4 % (65.3–94.8)	81.3 % (72.4–87.8)	85.3 % (70.1–93.5)
Lower Cost	65.7 % (45.2–81.7)	58.8 % (44.5–71.7)	63.0 % (49.7–74.6)
Easier to Access	58.7 % (39.3–75.6)	66.8 % (58.7–74.0)	72.7 % (58.0–83.7)
Safer	54.9 % (34.3–74.0)	39.5 % (30.0–49.8)	46.0 % (29.1–63.8)
More Traditional/Religious	37.9 % (21.1–58.0)	31.1 % (23.0–40.7)	28.9 % (14.8–48.7)
Modes of Healthcare Access			
Medical Doctors	99.4 % (94.8–99.9)	97.7 % (94.0–99.1)	94.7 % (88.4–97.6)
Family and Elders	53.0 % (33.0–72.1)	51.9 % (37.9–65.6)	53.7 % (38.0–68.6)
Traditional Healers	28.7 % (13.8–50.3)	5.10 % (2.03–12.2)	6.81 % (3.03–14.6)
Pharmacists	26.8 % (13.1–47.0)	21.5 % (13.4–32.6)	14.2 % (4.95–34.6)
Herbal Vendors	11.8 % (3.30–34.6)	4.29 % (12.8–13.4)	5.33 % (2.00–13.4)
Friends/Neighbors	10.6 % (4.40–23.3)	17.0 % (11.0–25.4)	17.2 % (8.21–32.6)
TM Use			
for Symptomatic Ailments	66.7 % (17.3–54.3)	33.9 % (25.2–43.9)	42.7 % (30.1–56.2)
for Chronic Diseases	49.6 % (28.6–70.6)	25.9 % (17.9–35.8)	26.3 % (14.3–43.4)
for Reproductive Illnesses	45.1 % (23.3–69.1)	19.7 % (11.6–31.3)	16.4 % (8.75–28.5)
for Malaria/Febrile Illnesses	64.0 % (44.1–79.9)	61.9 % (46.9–75.0)	46.8 % (31.4–62.8)
for Spiritual/traditional uses	16.1 % (8.71–27.9)	9.30 % (5.13–16.3)	4.69 % (1.48–13.8)
for Neurologic Illnesses	45.1 % (23.3–69.1)	19.7 % (11.6–31.3)	16.4 % (8.75–28.5)
for Urogenital Conditions	35.3 % (17.7–58.0)	15.8 % (9.39–25.5)	13.4 % (7.56–22.8)
for Cancers	11.1 % (3.86–28.3)	17.8 % (7.87–35.4)	7.64 % (3.16–17.3)
for Disease Prevention	5.17 % (1.75–14.3)	4.66 % (2.24–9.43)	6.99 % (2.77–16.5)
for Worms/Parasites	30.7 % (16.0–50.7)	8.36 % (5.04–13.6)	17.0 % (10.3–26.8)
Modes of TM Use			
Mix with water	91.8 % (82.7–96.3)	81.6 % (73.3–87.7)	84.8 % (75.5–91.0)
Drink as a tea	61.6 % (40.8–78.9)	55.5 % (42.6–67.7)	65.9 % (54.9–75.3)
Drink as a soup	59.6 % (39.4–77.0)	42.9 % (32.6–54.4)	47.5 % (37.6–57.5)
Chew from the plant	38.4 % (21.2–59.1)	56.3 % (46.1–66.1)	59.1 % (43.9–72.8)
Drink with milk	33.2 % (15.4–57.5)	20.7 % (14.5–28.5)	24.3 % (16.0–35.1)
Bath	33.5 % (14.6–59.7)	25.5 % (17.8–35.0)	31.8 % (16.8–51.9)
Inhalation	49.3 % (27.7–71.1)	30.2 % (20.0–42.8)	42.4 % (29.7–56.1)
Powders	19.5 % (2.85–48.5)	15.0 % (9.75–22.2)	26.5 % (12.4–47.9)
As foods to be eaten	2.40 % (0.51–10.6)	3.64 % (1.31–9.67)	2.96 % (0.76–10.8)
Pill/Vitamin form	2.59 % (0.60–10.5)	0.63 % (0.19–2.05)	0.85 % (0.20–3.46)
Lotions/Creams	1.21 % (0.26–5.50)	6.05 % (3.06–11.6)	7.81 % (3.10–18.3)

Chronic Diseases: Hypertension, Heart problems, Diabetes, or Body Swelling

Reproductive illnesses: Sexual Arousal/Virility, Menstrual Problems, Pregnancy Termination, or Fertility/Impotence

Neurologic illnesses: Epilepsy, Mental Confusion, or Depression

Spiritual/Traditional: Peace of mind/Ward off curses, Protection from ‘evil eyes’, Unexplained Illnesses, or ‘To Improve Luck’

Symptomatic Ailments: Increase Strength, Constipation, Increase energy, Digestion/Stomach problems, Fatigue, Arthritis/joint pains, Flu/Cold symptoms, Headaches, or Skin problems

Urogenital: Kidney problems or Urinary problems

Among those at increased risk for CKD, the prevalence of TM use was 49.0 % (95 % CI 33.1-65.0 %) with most adults using TMs 1–5 times (31.0 %; 95 % CI 20.8–43.3 %) or 6–10 times (11.5 %; 95 % CI 5.50–22.4 %) per year (Table 2). The incidence of TM use of more than ten times per year was 7.10 % (95 % CI 3.60–13.5 %). Among adults at risk for CKD, the prevalence of concurrent TM and biomedicine use was 10.4 % (95 % CI 4.70–21.6 %). In univariable regression, the ongoing use of biomedicines (PR = 1.77; *p* < 0.01) or having no formal education (PR = 2.10; *p* = 0.08) had the strongest associations with the use of TMs among adults at risk for CKD. There was no significant difference by age, gender, occupation, setting (urban/rural), ethnicity, or self-reported history of an NCD or HIV.

Modes of healthcare access were varied among adult community members with CKD. Nearly all reported seeking healthcare advice from medical doctors (99.4 %; 95 % CI 94.8–99.9), but other sources of healthcare were also prevalent including family and elders, traditional healers, pharmacists, herbal vendors, and friend/neighbors (Table 2). Modes of healthcare access were also varied among community members without CKD, but people with CKD were significantly more likely to report seeking advice from traditional healers than those at low risk (PR = 5.07; p < 0.01) or those at increased risk for CKD (PR 4.21; *p* = 0.01). They also tended toward a higher prevalence of obtaining healthcare advice from herbal vendors compared to those at low risk (PR 2.56; *p* = 0.20) or increased risk for CKD (PR 2.22; *p* = 0.29).

Adults with CKD reported using TMs for a variety of conditions/diseases (Table 2). TM was most prevalent for symptomatic ailments (66.7 %; 95 % CI 17.3–54.3), malaria/febrile illnesses (64.0 %; 95 % CI 44.1–79.9), chronic diseases (49.6 %; 95 % 28.6–70.6), reproductive illnesses (45.1 %; 95 % CI 23.3–69.1), and neurologic illnesses (45.1 %; 95 % CI 23.3.–69.1). Other frequent uses included, urogenital conditions, worms/parasite treatment, and spiritual/traditional reasons, and less commonly TMs were used for treating cancers and disease prevention. The most common modes were mixing with water (91.8 %; 95 % CI 82.7–96.3), drinking as a tea (61.6 %; 95 % CI 40.8–78.9), and drinking as a soup (59.6 %; 95 % CI 39.4–77.0). Other commons modes were inhalation, chewing straight from the plant, drinking with milk, and bathing.

### Traditional medicines used for the treatment of kidney disease in Kilimanjaro

In addition to the high prevalence of TM use among persons with CKD and at high risk for CKD, we identified many TMs in use among the general population specifically for the treatment of kidney diseases. We recorded 168 plant-based traditional medicines referenced by participants during the qualitative sessions. Five of these traditional medicines were referenced directly by participants as being used for local treatments of kidney disease: Aloe (*Aloe vera*), African Myrrh (*Commifora africana*), Lemongrass (*Cymbopogon citrullus*), avocado (*Persea americana*), and knob wood (*Zanthoxylum chalybeum*) (Table 3).

**Table 3:** Traditional medicines used for the local treatment of kidney diseases in Kilimanjaro Region, Tanzania

Nomenclature			Uses in other African communities	Active Compounds and Pharmacology	Plant Parts in Use	Potential Side Effects and Toxicities
Scientific	English Common Name	Local Vernacular				
*Aloe vera (ferox and secundiflora species)*[9–11, 13–18]	Cape aloes, Aloe Vera	Aloe	Southern Africa: arthritis, burns/skin conditions, hypertension, purging/laxative, GI upset/stomach aches, anti-inflammatory, cosmetics, eye ailments/conjunctivitis, sexually transmitted diseases, infertility, impotence	- Gel: Prostaglandin- and bradykinase-mediated anti-inflammatory activity.	Gel extract Leaves Rind Stem	- Volume depletion and electrolyte imbalance
				- Aloin leaf extracts: increases GI motility and induces emesis		- Hypoglycemia
						-Hyperpigmentation and photosensitivity
			East Africa (Kenya, Uganda, Ethiopia, and Tanzania): malaria, purging/laxative for cleansing purposes, GI upset/stomach aches, skin ulcerations/wound healing, cosmetic, infertility, anti-parasitic	Active compounds: glucomannans, thiamine, niacin, riboflavin, bradykinase, anthraquinone glycosides (aloin, barbaloin)		-Hepatotoxicity
						-Acute tubular necrosis
						-Acute interstitial nephritis
*Commifora africana* [19–23]	African myrrh, African bdelium (Hairy) Corkwood, Gumwood	Loduwa	Nigeria: Anti-helminthetic, Hypnotic/sedative, anti-epileptic	-Leaf extract: in-vitro inhibition of tumor cell proliferation and anti-oxidation	Stem Fruits Leaves Bark Resin	-GI upset including diarrhea and nausea
			Uganda: skins ulcerations/wound healing	-Resin: anti-parasitic activity		-Skin rashes (dermatitis)
			Southern Africa: Malaria/fever, Typhoid, skin ulcerations/wound healing, migraine, stomach aches	Active compounds: flavanoids, tannis, anthraquinone, cardiac glycosides, alkaloids		-Allergy and hypersensitivity reactions
						-Sedation/somnolence
*Cymbopogon citrullus* [12, 24–30]	Lemongrass	Mchaichai	South Africa: Diabetes, oral thrush	-Oil extracts have anti-bacterial, anti-amebic, anti-fungal, anti-malarial, anti-protozoal, and anti-filarial effects	Leaves Stem Oil extract	^-^Volume depletion
						-Diarrhea
						-Somnolence
						-Chronic kidney disease (decline in glomerular filtration rate)
			Mauritius: common cold, pneumonia, fever, GI upset/stomach aches	-Phenol and flavanoids have anti-oxidative properties		
						-Gastritis
						-Hepatotoxicty (potential)
			Nigeria: antipyretic/anti-malarial, stimulant, anti-spasmodic	-Citral has insect repellent properties		-Hypoglycemia
			Cameroon: anti-malarial, jaundice	Active compounds: Terpenes, alcohols, ketons, aldehyde, flavanoids, phenols, citral.		
			Angola: anti-tussive, anti-emetic, antiseptic, arthritis			
*Persea americana* [31–39]	Avocado	Mparachichi, Mpea, Mwembe, Mafuta	West Africa (Nigeria, Togo, Ivory Coast): anti-diarrheal, diabetes/hyperglycemia, anti-inflammatory, wound healing, anti-epileptic, exhaustion , hypertension, gastritis/dyspepsia	-Leaf extracts have direct vaso-dilatory properties	Leaves Fruits Seeds Rind Bark	-Increased risk of bleeding when combined with other anti-coagulants
				-Anti-inflammatory properties similar to acetylsalicylate and prostaglandin-inhibitors		-Hypoglycemia
			East Africa (Kenya, Uganda, Tanzania, Zimbabwe, Mozambique): dengue vector control, diarrhea, sore throat, menstrual regulation, hair growth, epilepsy, toothaches, wound healing, tuberculosis, neuralgia)	-Inhibits alpha-amylase and enhance glycogenesis		-Hyperkalemia (especially among those with impaired kidney function)
				-β-Carotene and fatty acids with lipid lowering properties		
				-Anti-convulsive effects possibly via gabanergic properties		
				-acetogenins inhibit platelet aggregation		
				-larvicidal to *Aedes aegypti*		
				Active Compounds: Tannins, saporins, alkanols (aliphatic acetogenins), terpenoids, coumarin		
*Zanthoxylum chalybeum* [19, 40–48]	Knob wood	Murungurungu, Simba Mwitu	Kenya and Zimbabwe: anti-malarial and anti-bacterial, snakebites, skins ulcerations/wound healing, arthritis	-Extracts from leaves, roots, and stems have prostaglandin-mediated anti-inflammatory activity	Root Stem Leaves Fruits Branches Seeds	-Anti-histamine effects including sedation
			Tanzania: swelling reduction, hernias, arthritis, asthma, arrow-tip poison	-Root bark has a fluroquinolone alkaloid with anti-bacterial and anti-malarial activity		-Gastritis/stomach ulcerations
			Uganda: dental caries/toothaches (chewed), anti-malarial/fevers, arthritis/joint aches, sickle cell disease	-Quaternary alkaloids have neuromuscular blocking effects (paralysis and tonic-contractions)		
				Active compounds: Skimmianine (flouroquinolone-alkaloid), tembetarine, nitidine, flavanoids, saponins		

## Discussion

The prevalence of TM use was high among persons with CKD and at risk for CKD in northern Tanzania. Additionally, the prevalence of concurrent use of TMs and biomedicines was high. Community members with CKD in northern Tanzania frequently sought healthcare advice from many non-biomedical practitioners including family members, community and tribal elders, friends, traditional healers, and herbal vendors. Cost, effectiveness, access, and safety were reported as common reasons for TM use over or concurrent with biomedicine. This is consistent with previous studies that have shown these to be important factors, especially among the NCDs, for the use of TM in Northern Tanzania [4,5].

The low disease awareness among persons with CKD implies that the high prevalence of TM use among this population may reflect their chronically poorer overall health or their perception of poorer health. Behind daily symptomatic ailments and malaria/febrile illness, the prevalence of TM use for chronic diseases was the highest which suggests that TM use may also be high for other NCDs such as diabetes and hypertension. In a region where CKD and NCD prevalence is high and many people continue to be at risk for these diseases, further understanding of TM practices surrounding NCDs will be important [4].

Separate from the use of TM among persons living with CKD, we studied the TMs used among the general population for the treatment of kidney disease. Although different disease understandings likely meant that what was called ‘kidney disease’ in a local context was quite different from the biomedical terms ‘chronic kidney disease’ or ‘acute kidney injury’ (e.g. urinary tract infections were frequently referred to as some form of kidney disease), understanding the TMs used for perceived kidney disease in any context remains important. Disease prevalence is high in the region, and public health programs aimed at increasing awareness need to be fully informed about their impact [4]. For example, many of the TMs used to treat what is perceived as kidney disease in the region do have pharmacologically active compounds with a wide-spectrum of effects including lipid-lowering, anti-microbial, anti-inflammatory, and anti-oxidative actions. However, we also found that they have multiple potential adverse effects including diarrhea, volume depletion, increased bleeding risk, hepatotoxicty, and nephrotoxicity, and these adverse effects may be augmented by the presence of CKD or their concurrent use with biomedicines. As such, CKD treatment programs and public health policies aimed at addressing CKD may have inadvertent consequences for patients and providers if they do not recognize these practices among their target populations.

Furthermore, much remains unknown about the causes of CKD in the region, and cataloguing the regional ethnopharmacology will be important in understanding and treating CKD [5]. It is not known to what extent TM use contributes to the prevalence or progression of CKD, but plant-based TMs have many mechanisms of nephrotoxicity including renal tubular damage, hypertension, papillary necrosis, acute tubular necrosis, interstitial nephritis, and nephrolithiasis [6,7]. We identified five plant-based TMs used for kidney disease in the region, two of which have direct potential nephrotoxicities: *Aloe vera* and *Cymbopogon citrullus* [10–12]. In many instances, this nephrotoxicity is dose-dependent, and this underscores the additional importance of also understanding the mode by which people consume TMs because people with CKD may be particularly vulnerable to these effects. In the case of *Aloe vera*, which can cause acute tubular necrosis and acute interstitial nephritis in addition to chronic renal insufficiency, the nephrotoxicy is substantially higher with the larger doses ingested by boiling and drinking the plant [10,11]. People with CKD may be particularly vulnerable to adverse effects from TMs, and as such, biomedical clinics caring for these populations may need to provide direct education about specific types and modes of TMs to avoid. As an example, a visual catalogue with pictures of the various forms of high-risk TMs may be useful as a guide for patients and providers (both biomedical and traditional).

Our study has many strengths. To our knowledge, this is one of few assessments of TM practices among a community-based, representative sample of participants with CKD, and because of our random sampling methods, these prevalence estimates may be generalizable across the regional population. Additionally, we also were able to assess TM use and practices among other community-based populations at risk for CKD. Finally, the qualitative sessions provided insight into the use of TMs for the local treatment of kidney disease and allowed us to explore and identify additional plant-based TMs used for treating kidney disease.

We also noted a few limitations. As this was a cross-sectional study, causal inferences cannot be drawn and associations may be influenced by confounding from unmeasured variables. Furthermore, our study may be subject to non-response bias, and to reduce this potential bias we used sample-balanced weights when reporting prevalence estimates. Our study may also have been subject to reporting and recall bias especially around the topics on frequencies of TM use. To reduce these biases, we used only local native surveyors who spoke Swahili as their first language, conducted the interviews in private when possible, and pre-tested the survey instrument for content validity and design flaws. Misclassification of disease may also be present, and though we expect most misclassification to be non-differential, the measurement we used to diagnose diabetes (HbA1c) has not been validated in this population. As such, the sensitivity and specificity of the test at a cutoff value of 7.0 % are not known for this population.

## Conclusion

In conclusion, the prevalence of TM use is high among adults with and at risk for CKD in northern Tanzania, and many of these same people use biomedicine and TM concurrently. People with CKD seek healthcare advice from many sources other than biomedical doctors such as traditional healers, herbal vendors, and family members, and they use TMs to treat a variety of conditions including other NCDs. The TMs commonly used for the local treatment of kidney disease have a wide range of activities, and people with CKD may be particularly vulnerable to adverse effects. Recognizing these traditional medicine practices will be important in shaping CKD treatment programs and public health policies aimed at addressing CKD.

## Additional file

Additional file 1: Appendix. Structured Survey Instrument for the use of Traditional Medicines (English and Swahili) (DOCX 461 kb)

## Abbreviations

CI: Confidence interval
CKD: Chronic kidney disease
CKD-AFRikA: Comprehensive Kidney Disease Assessment for Risk Factors, epidemiology, Knowledge, and Attitudes Study
COPD: Chronic obstructive pulmonary disease
eGFR: Estimated glomerular filtration rate
FGDs: Focus group discussions
HbA1C: Hemoglobin A1C
HIV: Human immunodeficiency virus
IQR: Inter-quartile range
KCMC: Kilimanjaro Christian Medical College
NCD: non-communicable disease
PR: Prevalence ratio
REDCap: Research Electronic Data Capture
TM: Traditional medicine
