# Supplementary material for: Traditional medicine practices among community members with chronic kidney disease in northern Tanzania: an ethnomedical survey
Source: BMC Nephrol. 2015 Oct 23;16:170. doi: 10.1186/s12882-015-0161-y (PMC4619231; doi:10.1186/s12882-015-0161-y)
Supplement: Additional file 1: Appendix. — Structured Survey Instrument for the use of Traditional Medicines (English and Swahili) (DOCX 461 kb) [file 12882_2015_161_MOESM1_ESM.docx]

**Traditional Medicines Practices Among Community Members with Chronic Kidney Disease in Northern Tanzania: An Ethnomedical Survey**

**Supplementary Appendix 1:**

**Structured Survey Instrument for the use of Traditional Medicines (English and Swahili)**

John W Stanifer*, MD, MSc^1,2^; Joseph Lunyera*, MBChB, MSc^2^; David Boyd, PhD ^2^; Francis Karia, MBA, MSc^3^; Venance Maro, MD, MMed^3^; Justin Omolo, PhD^4^; Uptal D Patel, MD^1,2,5^

For the

Comprehensive Kidney Disease Assessment for

Risk factors, epIdemiology, Knowledge, and Attitudes (CKD AFRIKA) Study

1 Department of Medicine, Duke University; Durham, NC United States

2 Duke Global Health Institute, Duke University; Durham, NC United States

3 Kilimanjaro Christian Medical College; Moshi, Tanzania

4 National Institute for Medical Research, Dar es Salaam, Tanzania

5 Duke Clinical Research Institute, Duke University; Durham, NC United States

*Co-first authors

Correspondence:

John W Stanifer, MD, MSc

Duke University Medical Center

Box 3182

Durham, NC United States 27710

Email: john.stanifer@duke.edu

Phone: 423 526 7113

Fax: 919 681 6448

**General Questions Related to Traditional Medications and Health**

**(*To be read to the participant):*** Traditional medications are important components of health and wellness. We are seeking to understand the nature and frequency of commonly used traditional medications. Traditional medications may include herbals, natural medicines, drugs, vitamins, teas, foods, creams, lotions, potions, and soups that are used to treat health and wellness problems. The responses you provide do not mean that you use traditional medications or that you believe in their use; rather, your responses will provide valuable information for addressing some of the unknown factors that influence access and costs of healthcare in this region.

1. What traditional medications have you heard about?

______________________________________________________________

1. Do you know anyone who uses any traditional medicines, natural medicines, vitamins, foods, teas, potions, or creams for health or wellness problems?

Yes No

If yes, how do you know him/her?

Family Member Friend Neighbor Other

What do they use? ________________________________

Cost is a big factor in people’s decision to use traditional medicines:

Yes No I do not know

Worry of side effects from hospital medicine is a big factor in people’s decision to use traditional medicines:

Yes No I do not know

People commonly use traditional medications and hospital medications at the same time.

Yes No I do not know

1. For what health reasons do people commonly use traditional medicines? Traditional medicines may include herbals, foods, drugs, vitamins, soups, teas, potions, or creams. (*Circle all that apply*)

| **Epilepsy** | **Sexual arousal/increase virility** | **Menstrual problems** | **High Blood Pressure** | **Increase strength** | **Kidney Problems** |
| --- | --- | --- | --- | --- | --- |
| **Mental confusion** | **Constipation** | **Pregnancy termination** | **Fever** | **Increase energy** | **Digestion/stomach problems** |
| **Heart problems** | **Depression** | **Infections** | **To stay healthy** | **HIV/AIDS** | **Fatigue** |
| **Arthritis (joint pains)** | **Worms/parasites** | **Flu/Cold symptoms** | **Fertility/Impotence** | **To prevent illness** | **Headaches** |
| **Urinary problems** | **Peace of mind/ward off curses** | **Protection from “evil” eyes** | **Unexplained illnesses** | **To improve health** | **To improve luck** |
| **Diabetes** | **Inside Cancers** | **Body Swelling** | **Malaria** | **Outside Cancers** | **Skin problems** |

Please write any additional uses:

1. How often do you think people use traditional medicines? Traditional medicines may include herbs, foods, soups, vitamins, teas, potions, or creams.

Never……………………………………………………………..

Everyday……………………………………………………..

1 to 5 times per month…………………………..

6 to 10 times per month………………………..

1 to 5 times per year……………………………….

1. In the past year, how many times did you use traditional medicines? Traditional medicines may include herbs, foods, inhalations, soups, vitamins, teas, potions, or creams.

None……………………………………………………………..

1 to 5 times …………………………………………..…..

6 to 10 times ………………………………….………..

11 to 20 times ………………………………………….

More than 20 times……………………………….…

1. When you have a health problem whom do you see for advice? *(circle all that apply)(place an asterisk by the one considered to be the most common)*

| **Family Member** | **Friends or Neighbors** | **MD (Doctor of Medicine)** |
| --- | --- | --- |
| **Tribal Chief or Elder** | **Village Elder or Leader** | **Wamaasai** |
| **Pastor or religious leader** | **Pharmacist** | **Traditional Healer** |

1. Why would you go to a hospital?

Diagnosis only

Treatment only

Diagnosis and Treatment

You would never go to a hospital

1. What are the most commonly used preparations or forms that people use? (*circle all that apply)*

| **Tea** | **Milk** | **Boiled Soup** |
| --- | --- | --- |
| **Lotion or Cream** | **Foods to be eaten (e.g. black chicken, rooster, goat, sheep)** | **Pill/vitamin** |
| **Water** | **Injection** | **Chewed or taken straight from the plant** |
| **Inhalation** | **Powders** | **Baths** |

Another way that is not listed:

____________________________________________________

1. Some persons commonly use traditional medicines and some persons commonly use prescription medicines as given by a Medical Doctor.

Why do people commonly use traditional medicines? Traditional medicines may include herbals, foods, drugs, soups, vitamins, teas, potions, or creams. (*circle all that apply*)

| **They are more affordable** | **Too hard to find a Medical Doctor** | **Failure of Hospital Medicines** |
| --- | --- | --- |
| **They are safer** | **They work better** | **Family tradition** |
| **Religious reasons** | **They are easier to obtain** | **They are more traditional** |
| **They are found naturally (free)** | **Hospital Medicines have too many chemicals** | **They have fewer side effects** |

**Comments for the Interviewer:**

**Maswali ya Jumla yanayohusiana na Matibabu ya Mitishamba na afya.**

**(*Isomwe kwa mshiriki):*** Mitishamba na dawa za asili ni kiungo muhimu katika afya na uzima.Tunatafuta kuelewa asili na mfululizo wa dawa zinazotumika kwa kawaida za mitishamba. Matibabu ya asili yanaweza kuhusisha mimea,dawa za kemikali,virutubisho/vitamin, chai, chakula, krimu, losheni, dawa inayosemekana kuwa na athari za kichawi, na supu vinavyotumika kutibu matatizo ya afya na uzima. Majibu unayotoa hayamaanishi kuwa unatumia matibabu ya mitishamba au unaamini katika matumizi yake; isipokuwa majibu yako yatatupa taarifa muhimu ili kushughulikia baadhi ya mambo mbalimbali yasiyojulikana yanayoathiri upatikanaji na gharama za matibabu katika mkoa huu.

1. Ni miti shamba ipi au dawa za asili umeshawahi kusika?

______________________________________________________________

1. Unafahamu mtu yeyote ambaye anatumia mitishamba,dawa za asili, vitamin, vyakula, chai, dawa zenye athari za kichawi au krimu kwa matatizo ya afya au uzima?

Ndiyo Hapana

Kama ndiyo, unamjuaje?

Mwanafamilia Rafiki Jirani Mwingine

Wanatumia nini? ________________________________

Gharama ni jambo kubwa sana katika maamuzi ya watu kutumia mitishamba au dawa za asili:

Ndiyo Hapana Sijui

Hofu ya madhara ya dawa za hospitali inachangia katika maamuzi ya watu kutumia mitishamba au dawa za asili.

Ndiyo Hapana Sijui

Je kwa kawaida watu hutumia dawa za hospitali na dawa za mitishamba kwa wakati mmoja?

Ndiyo Hapana Sijui

1. Ni sababu gani za kiafya ambazo watu kawaida hutumia mitishamba na dawa za asili? Dawa za asili zinaweza kuhusisha mimea, chakula, dawa za tiba, virutubisho/vitamin, krimu (*zungushia duara yote yanayohusika*).

| **Degedege** | **Hisia za kufanya mapenzi/Kuongeza nguvu za kiume** | **Matatizo ya Hedhi** | **Shinikizo la damu** | **Kuongeza uwezo** | **Matatizo ya figo** |
| --- | --- | --- | --- | --- | --- |
| **Kuchanganyikiwa** | **Choo ngumu** | **Kutoa mimba** | **Homa** | **Kuongeza nguvu** | **Umeng’enyaji chakula/Matatizo ya tumbo** |
| **Matatizo ya moyo** | **Msongo wa mawazo** | **Infections**  **Maambukizi** | **Kuwa na afya** | **Virusi vya Ukimwi/Ukimwi** | **Uchovu** |
| **Maumivu ya viungo** | **Minyoo** | **Mafua/ dalili za kifua** | **Uwezo wa kuzaa/Uhanithi** | **Kuzuia magonjwa** | **Kichwa kuuma** |
| **Matatizo ya kukojoa** | **Amani/kuondoa mikosi** | **Kujikinga na macho mabaya** | **Magonjwa yasiyoeleweka** | **Kuboresha afya** | **Kuongeza bahati** |
| **Kisukari** | **Saratani ya ndani ya mwili** | **Mwili kuvimba** | **Malaria** | **Saratani ya nje ya mwili** | **Matatizo ya Ngozi** |

Tafadhali andika matumizi yoyote ya nyongeza

________________________________________________________

1. Unafikiri ni kwa kiasi gani watu hutumia miti shamba au dawa za asili? Dawa za asili zinaweza kuhusisha mimea, chakula, dawa za tiba, vitamini au krimu.

Hawatumii……………………………………………………………..

Kila siku…………………………………………………………….…..

Mara1 hadi 5 kwa mwezi……………………….……..

Mara 6 hadi 10 kwa mwezi……………...…………..

Mara 1 hadi 5 kwa mwaka…………………….………….

1. Kwa mwaka uliopita ni mara ngapi umetumia dawa za mitishamba au dawa za asili?Dawa za asili zinaweza kuhusisha mimea, chakula, dawa za tiba, vitamin au krimu.

Haijatokea ……………………………………………………………..

Mara1hadi 5 ……………….………………………………………..

Mara 6 hadi 10 …………………………………………….……..

Mara 11 hadi 20 kwa mwezi …………………………….

Zaidi ya mara 20……………………………………………………

1. Ukiwa na matatizo ya kiafya huwa unamuona nani kutafuta ushauri? *(Zungushia yale yanayohusika)*

| **Mwanafamilia** | **Rafiki au Jirani** | **Daktari** |
| --- | --- | --- |
| **Chifu wa kabila au Mzee** | **Mzee wa Kijiji au Kiongozi** | **Wamasai** |
| **Mchungaji au kiongozi wa kidini** | **Mfamasia** | **Mganga wa mitishamba** |

1. Kwa nini unachagua kwenda hospitali?

Kupima tu

Kupata matibabu tu

Kupima na kupata matibabu

Siwezi kwenda hospitali kabisa

1. Ni aina gani ya matayarisho au namna ambayo kawaida watu huitumia? *(zungushia yote yanayotumika)*

| **Chai** | **Maziwa** | **Supu ya Kuchemshwa** |
| --- | --- | --- |
| **Losheni au Cream** | **Chakula chakuliwa**  **(Kuku mweusi,mbuzi wa kuchoma,kondoo)** | **Vidonge/Vitamini/virutubisho** |
| **Maji** | **Sindano** | **Kutafuna mizizi au kuchukua moja kwa moja kwenye mti** |
| **Kuvuta hewa** | **Unga** | **Kuoga** |

Namna nyingine ambayo haijaorodheshwa

____________________________________________________

1. Baadhi ya watu kawaida hutumia madawa ya asili na baadhi kawaida hutumia dawa walizoandikiwa na Daktari.

Kwa nini watu kawaida hutumia dawa za asili. Dawa za asili zinaweza kuhusisha mimea, chakula, dawa za tiba, vitamin au krimu.

*(Zungushia yote yanayotumika).*

| **Ni rahisi kuzimudu** | **Ni vigumu kumpata Daktari** | **Kutumia dawa za hospitali bila kupona** |
| --- | --- | --- |
| **Ni salama** | **Yanafanya kazi vizuri** | **Mila za kifamilia** |
| **Sababu za kidini** | **Ni rahisi kupatikana** | **Ni za kitamaduni zaidi** |
| **Yanapatikana zikiwa asilia (Bure)** | **Dawa za hospitali zina kemikali nyingi** | **Madhara kidogo kimwili** |

**Maoni ya mhojaji/mdahili:**
